# Podocyte-specific acid sphingomyelinase overexpression promotes gasdermin D dependent pyroptosis by impairing autophagic flux during obesity

**DOI:** 10.3389/fphys.2026.1856233

**Published:** 2026-07-29

**Authors:** Dandan Huang, Yao Zou, Ethan Chan, Jason M. Kidd, Xiaoyuan Wu, Yang Zhang, Todd W.B. Gehr, Ningjun Li, Pin-Lan Li, Guangbi Li

**Affiliations:** 1 Department of Biochemistry and Molecular Biology, School of Medicine, Virginia Commonwealth University, Richmond, VA, United States; 2 Department of Pharmacology and Toxicology, School of Medicine, Virginia Commonwealth University, Richmond, VA, United States; 3 Division of Nephrology, School of Medicine, Virginia Commonwealth University, Richmond, VA, United States; 4 Department of Pharmacological and Pharmaceutical Sciences, College of Pharmacy, University of Houston, Houston, TX, United States

**Keywords:** acid sphingomyelinase, autophagy, gasdermin D pore, lysosome, podocyte, pyroptosis

## Abstract

Recent studies suggest that gasdermin D (GSDMD) pore formation contributes to inflammasome-mediated cytokine release and pyroptosis in podocytes under pathological conditions. However, the molecular mechanisms regulating GSDMD pore formation in these cells remain unclear. Given the established role of the lysosomal acid sphingomyelinase (ASM)–ceramide pathway in obesity-related glomerulopathy (ORG), we investigated whether ASM regulates obesity-induced GSDMD pore formation and pyroptosis in podocytes, thereby influencing the progression of ORG. We found that podocyte-specific Smpd1 (the gene encoding ASM) overexpression markedly enhanced high-fat diet (HFD)-induced NLRP3 inflammasome activation, GSDMD N-terminal fragment (GSDMD-NT) generation, and pyroptosis in glomeruli of Smpd1^trg^/Podo^cre^ mice compared to wild-type controls. Pharmacological inhibition of ASM or the NLRP3 inflammasome attenuated these pathological changes in obese mice. In contrast, inhibition of GSDMD pore formation with disulfiram (DIS) prevented HFD-induced pyroptosis without affecting NLRP3 inflammasome activation. Consistently, obesity-induced podocyte injury and glomerulosclerosis were exacerbated by ASM overexpression but alleviated by inhibition of ASM, the NLRP3 inflammasome, or GSDMD pore formation. Using primary podocytes isolated from wild-type, Smpd1 knockout (Smpd1^-/-^), and Smpd1^trg^/Podo^cre^ mice, we further demonstrated that palmitic acid (PA), an obesity-associated lipotoxic factor, induced NLRP3 inflammasome activation, GSDMD pore formation, inflammasome product release, and pyroptosis. These responses were suppressed by Smpd1 deletion but enhanced by ASM overexpression. Confocal and super-resolution microscopy revealed that PA increased the accumulation of autophagosomes containing GSDMD-NT while impairing lysosome–autophagosome fusion, effects that were mitigated by Smpd1 deletion and amplified by ASM overexpression. To further elucidate the underlying mechanism, we examined whether ASM regulates lysosomal TRPML1 channel-mediated Ca^2+^ release, thereby controlling lysosome–autophagosome interaction and GSDMD-NT degradation. PA inhibited TRPML1 channel activity in podocytes, an effect that was intensified by ASM overexpression. Furthermore, PA-induced impairment of lysosome–autophagosome interaction and increased GSDMD pore formation were attenuated by the TRPML1 agonist ML-SA5 and exacerbated by the TRPML1 inhibitor ML-SI1. Collectively, these findings indicate that ASM regulates lysosomal function and autophagic degradation of GSDMD-NT, thereby controlling GSDMD pore formation and pyroptosis in podocytes during ORG.

## Introduction

Increasing evidence indicates that obesity is characterized by low-grade chronic systemic inflammation and oxidative stress (Matsuzawa-Nagata et al., 2008; Marseglia et al., 2014; McMurray et al., 2016; Alcala et al., 2017; Bayliak et al., 2019). Recent studies have further demonstrated that obesity-associated glomerular inflammation and the subsequent development of obesity-related glomerulopathy (ORG) are initiated by oxidative activation of the NLRP3 inflammasome in podocytes. This inflammasome complex, composed of NOD-like receptor protein 3 (NLRP3), the adaptor protein apoptosis-associated speck-like protein containing a caspase recruitment domain (ASC), and caspase-1, serves as a key inflammatory signaling platform (Martinon et al., 2009; Abais et al., 2013; Boini et al., 2016). Activation of the NLRP3 inflammasome leads to proteolytic processing of pro-inflammatory mediators, including interleukin-1β (IL-1β), interleukin-18 (IL-18), and high-mobility group box 1 (HMGB1) (Cruz et al., 2007; Halle et al., 2008; Nour et al., 2009; He et al., 2015). These inflammasome-derived factors play essential roles in initiating sterile inflammatory responses, ultimately contributing to glomerular inflammation and sclerosis during obesity (Martinon et al., 2002; Srinivasula et al., 2002; Chen and Nunez, 2010; Lamkanfi, 2011; Boini et al., 2014; Boini et al., 2016).

Despite these advances, the mechanisms by which NLRP3 inflammasome products are released from podocytes to propagate inflammatory signaling remain poorly understood. Emerging evidence indicates that gasdermin D (GSDMD) pore formation is required for IL-1β secretion and pyroptosis, a highly inflammatory form of regulated cell death, following NLRP3 inflammasome activation in multiple cell types (He et al., 2015; Dubois et al., 2019; Gao et al., 2021; Tsuchiya et al., 2021). In obesity-associated conditions such as non-alcoholic fatty liver disease and hepatocellular carcinoma, GSDMD pores have been shown to play critical roles in mediating inflammasome product release and pyroptosis (Rodriguez-Antonio et al., 2021; Yamagishi et al., 2022). Similarly, in glomerular diseases—including APOL1-associated podocytopathy (Wu et al., 2021a), lupus nephritis (Cao et al., 2021), membranous nephropathy (Lv et al., 2022), and diabetic nephropathy (Cheng et al., 2021)—increased GSDMD cleavage and pyroptosis have been observed in podocytes. However, comprehensive studies defining the activation and regulation of GSDMD pore formation in podocytes are still lacking. These observations raise the possibility that GSDMD pore formation represents a key mechanism underlying NLRP3 inflammasome-mediated cytokine release and pyroptosis in podocytes, thereby contributing to chronic glomerular inflammation, podocyte loss, and progression to focal segmental glomerulosclerosis (FSGS) in ORG.

Acid sphingomyelinase (ASM) and its downstream product ceramide have been implicated in the development of glomerular injury during obesity (Boini et al., 2010b; Boini et al., 2016). Ceramide-mediated glomerular damage has been attributed, at least in part, to increased reactive oxygen species (ROS) production and subsequent activation of the NLRP3 inflammasome in podocytes, leading to inflammatory responses (Boini et al., 2014; Boini et al., 2016). In addition, ceramide has been reported to induce GSDMD cleavage and pyroptosis in endothelial cells (Liu et al., 2022). Notably, ceramide and related sphingolipids are recognized as key regulators of lysosomal trafficking and membrane fusion processes across various cell types (Lee et al., 1998; Alvarez-Erviti et al., 2011; Lee et al., 2012; Liebau et al., 2013; Li et al., 2013; Lorber, 2014; Cui et al., 2016). In podocytes, recent studies have shown that acid ceramidase deficiency, which leads to ceramide accumulation, impairs lysosomal transient receptor potential mucolipin 1 (TRPML1) channel-mediated Ca^2+^ release, resulting in defective lysosomal trafficking and accumulation of autophagosomes (APs) and multivesicular bodies (MVBs) (Li et al., 2020a; Li et al., 2023). Furthermore, ASM overexpression and ceramide accumulation have been reported to disrupt lysosome–MVB fusion and thereby enhance exosome release in podocytes (Huang et al., 2021; Huang et al., 2022). However, whether lysosomal function regulates GSDMD pore formation and pyroptosis in podocytes under pathological conditions remains unknown.

In the present study, we hypothesized that the ASM–ceramide signaling pathway determines GSDMD pore formation and pyroptosis by modulating lysosomal function in podocytes. To test this hypothesis, we examined the effects of podocyte-specific ASM overexpression, as well as pharmacological inhibition of ASM, NLRP3 inflammasome activation, or GSDMD pore formation, on obesity-induced inflammasome activation, GSDMD cleavage, pyroptosis, podocyte injury, and glomerular sclerosis. Mechanistically, our *in vitro* studies further investigated whether ASM regulates GSDMD pore formation and pyroptosis through autophagy-dependent pathways. Our findings demonstrate that ASM plays a critical role in controlling GSDMD pore formation and pyroptosis in podocytes during obesity. These results suggest that targeting the autophagic degradation of GSDMD-NT in podocytes may represent a novel therapeutic strategy for preventing or treating glomerular inflammation and injury in a range of pathological conditions, including obesity, hyperhomocysteinemia, and diabetes mellitus.

## Materials and methods

### Animals

Podocyte-specific Cre recombinase mice (Podo^cre^; B6.Cg-Tg(NPHS2-Cre)295Lbh/J, stock no. 008205) were obtained from The Jackson Laboratory. Smpd1^trg^ mice carrying a floxed STOP cassette positioned downstream of the β-actin promoter and upstream of mouse Smpd1 cDNA were generously provided by Dr. Erich Gulbins (University of Duisburg-Essen, Essen, Germany). Eight-week-old male WT/WT and Smpd1^trg^/Podo^cre^ mice were used in all experiments. Animals were maintained on either a low-fat control diet (ND; D12450B, 10 kcal% fat) or a high-fat diet (HFD; D12492, 60 kcal% fat) obtained from Research Diets for a duration of 12 weeks (Huang et al., 2023). During the feeding period, mice receiving HFD were administered intraperitoneal injections every other day with vehicle, amitriptyline (10 mg/kg) (Xia et al., 2019; Huang et al., 2022), MCC950 (20 mg/kg) (Jiang et al., 2020; Jiao et al., 2020), or disulfiram (50 mg/kg) (Hu et al., 2020; Wu et al., 2021b). All animal procedures were conducted in accordance with protocols approved by the Institutional Animal Care and Use Committee at Virginia Commonwealth University (protocol AM10174). Female mice were not included in this study because prior reports have shown sex-dependent differences in metabolic responses to high-fat diet exposure, including distinct compensatory energy expenditure mechanisms between males and females (Maric et al., 2022). In addition, epidemiological data indicate that the prevalence of overweight and obesity varies by sex, with women generally exhibiting lower rates of overweight compared with men (Shah et al., 2020).

### Immunohistochemistry

Kidney tissues were processed for paraffin embedding, sectioned at 5 µm, and mounted on glass slides. Antigen retrieval was performed using heat-based methods, after which endogenous peroxidase activity was quenched by incubation with 3% hydrogen peroxide. To reduce nonspecific binding, sections were incubated with fetal bovine serum for 30 minutes. Sections were then exposed overnight at 4 °C to primary antibodies against cleaved caspase-1 (1:100; Cell Signaling Technology, Danvers, MA) or cleaved IL-1β (1:100; Medical and Biological Laboratories, Tokyo, Japan), prepared in PBS supplemented with 4% fetal bovine serum. After rinsing with PBS, sections were incubated with biotinylated IgG (1:200) for 1 hour at room temperature, followed by streptavidin–HRP for 30 minutes. Chromogenic detection was achieved using DAB for 1 minute, and nuclei were counterstained with hematoxylin for 5 minutes. Slides were mounted and examined under a light microscope. The extent of positive staining was quantified using Image-Pro Plus 6.0 software (Media Cybernetics, Bethesda, MD) (Li et al., 2020b).

### Immunofluorescent staining

Frozen sections of mouse kidney tissue were fixed with acetone and subjected to a blocking step to minimize nonspecific interactions. Samples were incubated overnight at 4 °C with primary antibodies against GSDMD-NT (1:100; ABclonal Technology, Woburn, MA) and podocin (1:200; Sigma-Aldrich, St. Louis, MO). Following washing, sections were incubated for 1 hour at room temperature with Alexa Fluor 488–conjugated secondary antibodies (Invitrogen, Carlsbad, CA). After additional washes, slides were mounted and visualized using a confocal laser scanning microscope (FluoView FV1000, Olympus, Tokyo, Japan). Fluorescence signals were quantified using ImageJ software (NIH, Bethesda, MD) (Huang et al., 2023).

### Glomerular morphological examination

Kidney tissues were fixed, embedded in paraffin, sectioned, and stained using periodic acid–Schiff (PAS). For each sample, 50 glomeruli per slide were evaluated under a light microscope by an investigator blinded to the experimental groups. Glomerular injury was graded on a scale of 0 to 4 based on the extent of sclerosis, defined as follows: 0, no detectable lesion; 1, sclerosis involving <25% of the glomerular area; 2, 25–50% involvement; 3, 50–75% involvement; and 4, >75% involvement. The severity of glomerular damage was quantified as the glomerular damage index (GDI), calculated using the formula ((N1 × 1) + (N2 × 2) + (N3 × 3) + (N4 × 4))/n, where N1–N4 correspond to the number of glomeruli assigned to grades 1 through 4, respectively, and n represents the total number of glomeruli evaluated (Abais et al., 2014).

### Primary culture of murine podocytes

Primary podocytes were isolated from mice as previously described (Li et al., 2020a; Huang et al., 2022; Huang et al., 2023; Li et al., 2023). Briefly, Dynabeads (20 mL) were perfused via the abdominal aorta below the renal artery at a rate of 7.4 mL/min/g kidney. Following perfusion, kidneys were excised, decapsulated, and the cortex was dissected and minced into small fragments. Tissue fragments were enzymatically digested in Hanks’ balanced salt solution containing collagenase A (1 mg/mL) and DNase I (0.2 mg/mL) at 37 °C for 20 minutes with gentle agitation. The digested suspension was passed through a 100 µm strainer and gently pressed with ice-cold medium. Glomeruli were washed six times with cold PBS, resuspended in culture medium, and plated onto collagen I-coated flasks. After 3 days, cellular outgrowths were detached using trypsin–EDTA and transferred to a glass tube. Magnetic separation was performed for 1 minute to remove bead-containing glomerular cores. The supernatant was then filtered through a 40 µm mesh to eliminate residual fragments. Isolated podocytes were cultured on collagen I-coated flasks in DMEM/F-12 (1:1) supplemented with 10% fetal bovine serum (Cansera International, Canada), 0.5% insulin–transferrin–selenium-A (Invitrogen), 100 U/mL penicillin, and 100 mg/mL streptomycin at 37 °C. For experiments, podocytes were exposed to palmitic acid (PA) at 200 μM for 24 hours, with treatment conditions selected based on prior studies (Ku et al., 2019; Wajngarten and Silva, 2019; Chobufo et al., 2020; Poznyak et al., 2022).

### Confocal microscopy

Podocytes cultured on collagen-coated glass coverslips were subjected to double immunofluorescence staining following experimental treatments and fixation. Cells were incubated overnight at 4 °C with primary antibodies, including goat anti-NLRP3 (1:100; Abcam Biotechnology, Cambridge, MA), rabbit anti-ASC (1:100; MilliporeSigma, Burlington, MA), rabbit anti-LC3 (1:100; Cell Signaling Technology, Danvers, MA), and rabbit anti-GSDMD-NT (1:100; ABclonal Technology, Woburn, MA). After washing, appropriate secondary antibodies were applied for 1 hour at room temperature, including Alexa Fluor 488–conjugated anti-goat (1:200), Alexa Fluor 594–conjugated anti-rabbit (1:200), Alexa Fluor 488–conjugated anti-rabbit (1:200), and Alexa Fluor 594–conjugated anti-rat (1:200) (Life Technologies, CA). Nuclei were labeled with DAPI. Samples were mounted and imaged using a confocal laser scanning microscope (Fluoview FV1000, Olympus, Japan). Colocalization analysis was conducted using Image-Pro Plus 6.0 software and expressed as Pearson correlation coefficients (Li et al., 2019; Li et al., 2020a).

### Structured illumination microscopy

Following treatment and fixation, cells were incubated overnight at 4 °C with rabbit anti-LC3 antibody (1:100; Cell Signaling Technology, Danvers, MA) and rat anti-LAMP-1 antibody (1:100; Santa Cruz Biotechnology, Dallas, TX). After washing, cells were incubated with Alexa Fluor 488–conjugated anti-rabbit and Alexa Fluor 594–conjugated anti-rat secondary antibodies (both 1:200; Life Technologies, CA) for 1 hour at room temperature. Cells were then washed, counterstained with DAPI, and mounted. Imaging was performed using a Nikon fluorescence microscope operating in structured illumination microscopy (SIM) mode. Colocalization was quantified using Image-Pro Plus 6.0 software and reported as Pearson correlation coefficients (Rozenova et al., 2010).

### Lactate dehydrogenase cytotoxicity assay

The lactate dehydrogenase (LDH) concentration in cell culture medium was measured to quantify cellular cytotoxicity (Ousingsawat et al., 2018). After treatments, supernatants were collected and measured using CyQUANT LDH cytotoxicity assay kit (Thermo Fisher Scientific, Waltham, MA) at wavelength of 490 nm. Percentage of cytotoxicity was calculated as (compound-treated LDH activity - spontaneous LDH activity)/(maximum LDH activity - spontaneous LDH activity) × 100.

### Flow cytometric analysis of pyroptosis

After treatments, podocytes were collected by trypsinization and centrifuged 500 × g 4 °C for 10 min. Cell pellets were washed with cold PBS and stained with FITC-labeled annexin V and 7-AAD for 10 min at room temperature in the dark. The fluorescence of annexin V-FITC and 7-AAD in at least 10,000 podocytes was immediately analyzed by flow cytometry using a flow cytometer (GUAVA, Hayward, CA). The percentage of podocytes positive of both annexin V-FITC and 7-AAD stainings was used to represent the pyroptotic cell death in podocytes (Ousingsawat et al., 2018).

### GCaMP3 Ca^2+^ imaging

Podocytes were used for imaging experiments 18–24 hours after nucleofection with the GCaMP3-ML1 construct (Li et al., 2019; Li et al., 2023). Changes in fluorescence were monitored at an excitation wavelength of 470 nm (F470) using a Nikon Diaphoto TMD inverted fluorescence microscope. Image capture, processing, and storage for subsequent analysis were carried out with Metafluor imaging software (Universal Imaging, Bedford Hills, NY). Lysosomal Ca^2+^ release was measured under ‘low’ extracellular calcium conditions using a solution composed of 145 mM NaCl, 5 mM KCl, 3 mM MgCl_2_, 10 mM glucose, 1 mM EGTA, and 20 mM HEPES (pH 7.4). To activate TRPML channels, cells were treated with the agonist ML-SA5 (1 µM), while ionomycin (1 µM) was applied as a positive control to induce maximal Ca^2+^ release.

#### Dynamic analysis of lysosome movement in podocytes

Podocytes cultured in 35 mm dish were incubated with 100 mg/ml dextran–Alexa Fluor 555 (Thermo Fisher Scientific, Waltham, MA, USA) for 6 h. The confocal fluorescent microscopic recording was conducted with a confocal laser scanning microscope (Fluoview FV1000, Olympus, Japan). The fluorescent images for lysosomes in podocytes were continuously recorded at an excitation/emission (nm) of 555/565 by using XYT recording mode with a speed of 1 frame/10 second for 10 min. Lysosome tracking was performed in Image J using manual tracking plugin. Ten lysosomes were chosen at random for each cell. These lysosomes were then tracked manually, while the program calculated velocity of lysosome trafficking for each frame (Li et al., 2022; Li et al., 2023).

### Statistical analysis

All statistical analyses were performed using SigmaPlot 14.0. Data are presented as mean ± SEM. Differences among multiple groups were evaluated by one-way ANOVA followed by the Student–Newman–Keuls *post hoc* test. A value of P < 0.05 was considered to indicate statistical significance.

## Results

### Enhancement of obesity-induced NLRP3 inflammasome activation in glomeruli by podocyte-specific Smpd1 gene overexpression

**Figure 1:**
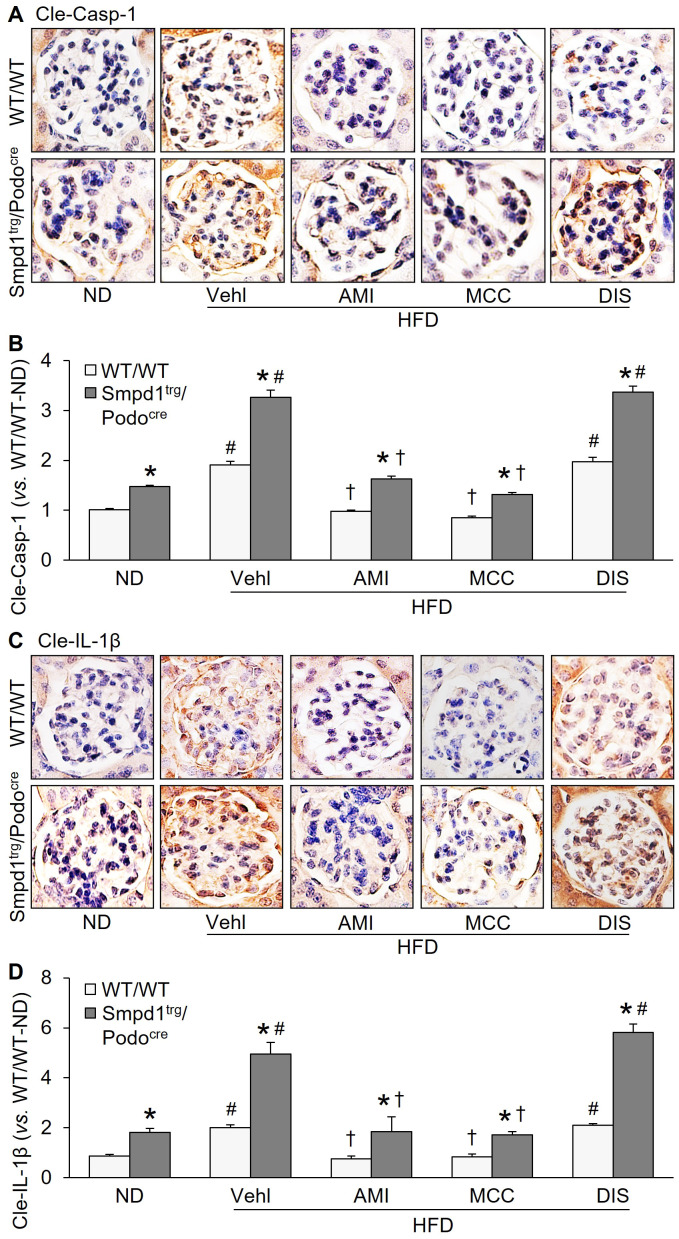
Enhancement of obesity-induced NLRP3 inflammasome activation in glomeruli by podocyte-specific Smpd1 gene overexpression. **(A)** Representative images showing the glomerular expression of cleaved caspase-1 in different groups of mice. **(B)** Summarized data showing the glomerular expression of cleaved caspase-1 in different groups of mice (n=6). **(C)** Representative images showing the glomerular expression of cleaved IL-1β in different groups of mice. **(D)** Summarized data showing the glomerular expression of cleaved IL-1β in different groups of mice (n=3-6). * P<0.05 *vs.* WT/WT group. # P<0.05 *vs.* ND group. † P<0.05 *vs.* Vehl group. ND, normal diet; HFD, high-fat diet; Vehl, vehicle; AMI, amitriptyline; MCC, MCC950; DIS, disulfiram; Cle-Casp-1, cleaved caspase-1; Cle-IL-1β, cleaved interleukin-1β.

To test our hypothesis, WT/WT and Smpd1^trg^/Podo^cre^ (podocyte-specific Smpd1 gene overexpression) mice were fed either a low-fat diet (normal diet, ND) or a high-fat diet (HFD) for 12 weeks (Huang et al., 2023). In parallel, HFD-fed mice received intraperitoneal injections of vehicle, amitriptyline (AMI; an ASM inhibitor), MCC950 (MCC; an NLRP3 inflammasome inhibitor), or disulfiram (DIS; a GSDMD pore formation inhibitor) every other day. Immunohistochemical staining for cleaved caspase-1, the active form of caspase-1 following NLRP3 inflammasome activation, and IL-1β was performed to evaluate whether obesity-induced inflammasome activation in glomeruli was affected by podocyte-specific ASM overexpression and pharmacological interventions. As shown in **Figures 1A, B**, glomerular levels of cleaved caspase-1 were minimal in WT/WT mice fed ND. HFD feeding significantly increased cleaved caspase-1 expression in glomeruli of WT/WT mice, and this effect was further amplified in Smpd1^trg^/Podo^cre^ mice. In contrast, the HFD-induced increase in cleaved caspase-1 in both WT/WT and Smpd1^trg^/Podo^cre^ mice was effectively suppressed by treatment with amitriptyline or MCC950, but not by disulfiram. We further examined the maturation of IL-1β by immunohistochemical staining for cleaved IL-1β, the active form generated downstream of caspase-1 activation. Consistent with the changes in cleaved caspase-1, glomerular cleaved IL-1β expression was low in WT/WT mice fed ND but was markedly increased by HFD feeding. Podocyte-specific Smpd1 overexpression further enhanced HFD-induced glomerular cleaved IL-1β expression in Smpd1^trg^/Podo^cre^ mice compared with obese WT/WT mice. The HFD-induced increase in glomerular cleaved IL-1β in both genotypes was attenuated by amitriptyline or MCC950, whereas disulfiram had no significant effect (**Figures 1C, D**). These results demonstrate that HFD induces NLRP3 inflammasome activation in glomeruli, as evidenced by increased cleaved caspase-1 and cleaved IL-1β, and that this inflammasome activation is enhanced by podocyte-specific Smpd1 gene overexpression. The inhibitory effects of amitriptyline and MCC950, but not disulfiram, further indicate that ASM acts upstream of NLRP3 inflammasome activation, whereas disulfiram acts downstream at the level of GSDMD pore formation.

### Exaggeration of GSDMD cleavage and pyroptosis in glomeruli of obese mice by podocyte-specific Smpd1 gene overexpression

**Figure 2:**
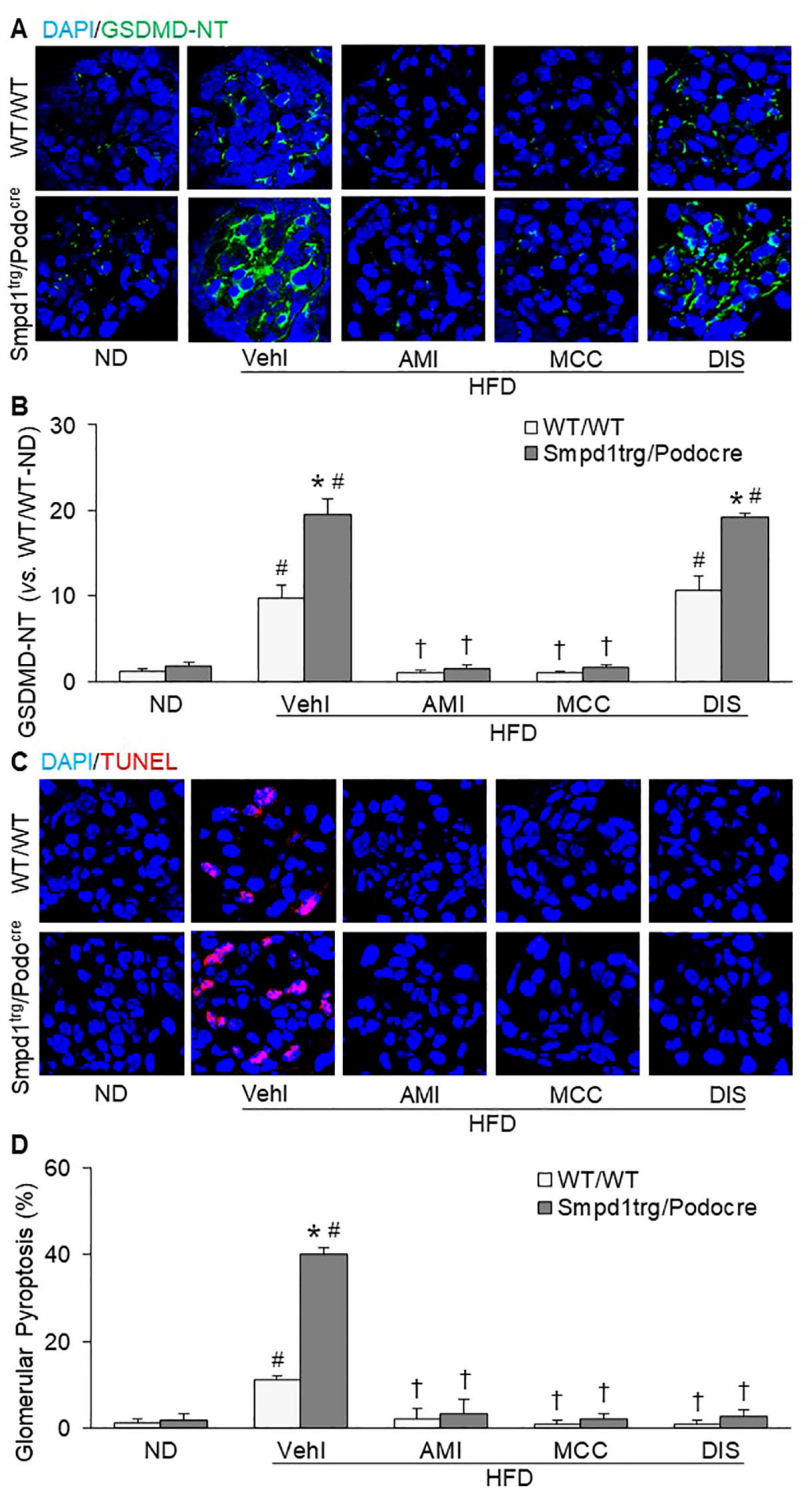
Exaggeration of GSDMD cleavage and pyroptosis in glomeruli of obese mice by podocyte-specific Smpd1 gene overexpression. **(A)** Representative images showing the glomerular expression of GSDMD-NT in different groups of mice. **(B)** Summarized data showing the glomerular expression of GSDMD-NT in different groups of mice (n=4-5). **(C)** Representative images showing the pyroptotic cell death in glomeruli detected by TUNEL assay. **(D)** Summarized data showing the percentages of pyroptotic cell death in glomeruli detected by TUNEL assay (n=3-6). * P<0.05 *vs.* WT/WT group. # P<0.05 *vs.* ND group. † P<0.05 *vs.* Vehl group. ND, normal diet; HFD, high-fat diet; Vehl, vehicle; AMI, amitriptyline; MCC, MCC950; DIS, disulfiram; GSDMD-NT, gasdermin D N-terminal.

Given the critical role of GSDMD pore formation in the release of NLRP3 inflammasome products (He et al., 2015; Dubois et al., 2019; Gao et al., 2021; Tsuchiya et al., 2021), we examined GSDMD cleavage in glomeruli by assessing the GSDMD N-terminal fragment (GSDMD-NT) using immunofluorescence staining. As shown in **Figures 2A, B**, GSDMD-NT levels were minimal in glomeruli of WT/WT mice maintained on a ND. HFD feeding significantly increased GSDMD-NT expression in WT/WT mice, and this effect was further enhanced in Smpd1^trg^/Podo^cre^ mice with podocyte-specific ASM overexpression. In contrast, the HFD-induced increase in glomerular GSDMD-NT in both genotypes was abolished by intraperitoneal administration of amitriptyline or MCC950, whereas disulfiram treatment had no effect. These results are consistent with the expected pharmacological actions of these inhibitors, because amitriptyline and MCC950 act upstream of GSDMD cleavage by inhibiting ASM activity and NLRP3 inflammasome activation, respectively, whereas disulfiram acts downstream by inhibiting GSDMD pore formation rather than preventing GSDMD cleavage.

**Figure 3:**
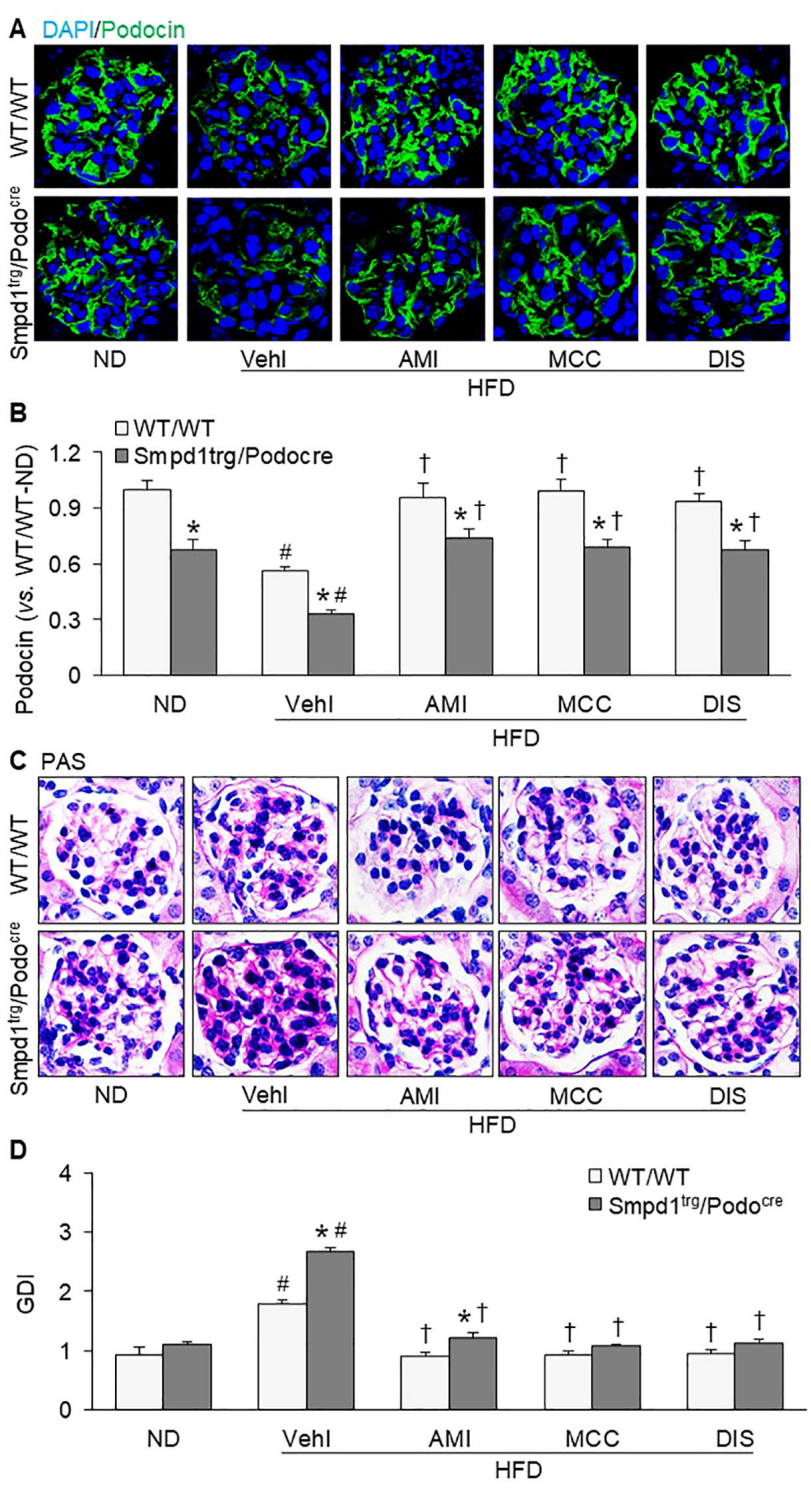
GSDMD pore formation prevented by Smpd1 gene deletion in podocytes. **(A)** Representative images showing the colocalization of NLRP3 and ASC in different groups of podocytes. DAPI was used to stain nuclei. **(B)** Summarized data showing the colocalization of NLRP3 and ASC in different groups of podocytes (n=3-4). **(C)** Representative images showing the colocalization of GSDMD-NT and WGA on plasma membrane of different groups of podocytes. DAPI was used to stain nuclei. **(D)** Summarized data showing the colocalization of GSDMD-NT and WGA on plasma membrane of different groups of podocytes (n=3-4). **(E)** IL-1β release from different groups of podocytes (n=5-8). * P<0.05 *vs.* Vehl group. # P<0.05 *vs.* WT/WT group. Vehl, vehicle; PA, palmitic acid; GSDMD-NT, gasdermin D N-terminal.

**Figure 4:**
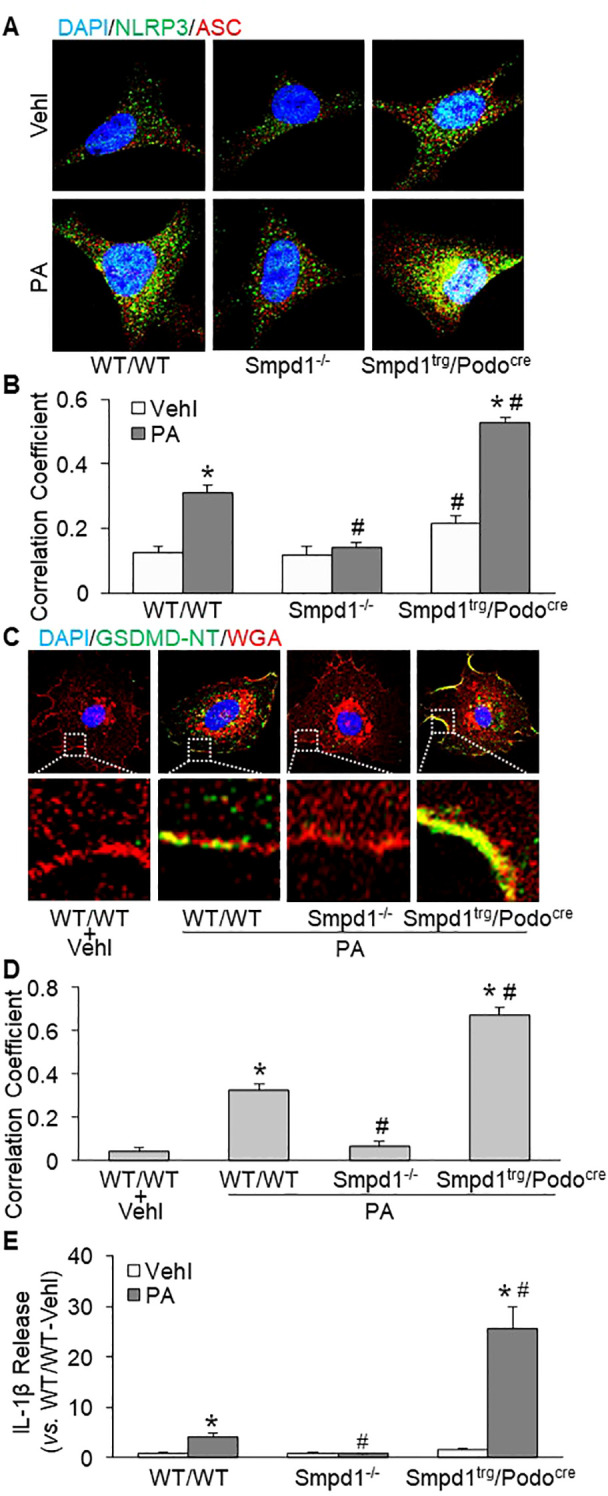
GSDMD pore-mediated pyroptosis is regulated by ASM activity in podocytes. **(A)** LDH release from WT/WT podocytes treated with vehicle or increasing concentrations of PA for 24 hours. LPS plus nigericin was included as a positive control for canonical pyroptosis (n=3-4). **(B)** LDH release from WT/WT podocytes treated with PA in the absence or presence of disulfiram (DIS), Z-DEVD-FMK (DEVD), GW806742X (GW), or Ferrostatin-1 (FER) (n=3-8). **(C)** LDH release from WT/WT, Smpd1^-/-^, and Smpd1^trg^/Podo^cre^ podocytes treated with vehicle or PA (n=5-8). **(D)** Representative dot plots of flow cytometric analysis of Annexin V-FITC and 7-AAD staining in different groups of podocytes. **(E)** Summarized data showing the percentages of Annexin V-FITC/7-AAD double-positive podocytes in different groups (n=6). *P<0.05 *vs.* Vehl group. **P<0.001 *vs.* Vehl group. # P<0.001 *vs.* PA group. †P<0.001 *vs.* WT/WT group. PA, palmitic acid; DIS, disulfiram; DEVD, Z-DEVD-FMK; GW, GW806742X; FER, Ferrostatin-1; 7-AAD, 7-aminoactinomycin D.

We next evaluated pyroptotic cell death in glomeruli using a TUNEL assay kit (Cell Signaling Technology, Danvers, MA). Consistent with the GSDMD-NT results, podocyte-specific Smpd1 overexpression markedly exacerbated HFD-induced pyroptosis in Smpd1^trg^/Podo^cre^ mice compared with obese WT/WT controls. Pharmacological inhibition with amitriptyline, MCC950, or disulfiram effectively prevented pyroptotic cell death in glomeruli of both WT/WT and Smpd1^trg^/Podo^cre^ mice during obesity (**Figures 2C, D**). The protective effect of disulfiram, despite persistent GSDMD-NT generation, suggests that inhibition of GSDMD pore formation is sufficient to suppress pyroptosis in glomeruli downstream of inflammasome activation and GSDMD cleavage. Together with our *in vitro* findings in primary podocytes (**Figures 3**, **4**), these *in vivo* data support that HFD induces GSDMD-associated pyroptosis in glomeruli and that this response is exaggerated by podocyte-specific Smpd1 overexpression.

### Attenuation of obesity-induced glomerular injury by inhibition of GSDMD pore formation

We next investigated whether GSDMD pore formation contributes to obesity-induced podocyte injury and glomerular sclerosis. As shown in **Figures 3A, B**, WT/WT mice fed a HFD exhibited a marked reduction in glomerular podocin expression, a key structural protein of the podocyte slit diaphragm, compared with control littermates. This loss of podocin was further exacerbated in Smpd1^trg^/Podo^cre^ mice with podocyte-specific Smpd1 overexpression. In contrast, intraperitoneal treatment with amitriptyline, MCC950, or disulfiram significantly attenuated the HFD-induced decrease in podocin expression in both genotypes. We further assessed morphological and structural alterations in glomeruli among the experimental groups. Consistent with the changes in podocin expression, PAS staining revealed prominent sclerotic lesions in glomeruli of HFD-fed WT/WT mice, which were more severe in Smpd1^trg^/Podo^cre^ mice. Notably, administration of amitriptyline, MCC950, or disulfiram effectively prevented the development of glomerular sclerosis in both WT/WT and Smpd1^trg^/Podo^cre^ mice (**Figures 3C, D**).

### PA-induced pyroptosis is mediated primarily by GSDMD pore formation and regulated by ASM activity in podocytes

For *in vitro* studies, we isolated podocytes from WT/WT, Smpd1^-/-^, and Smpd1^trg^/Podo^cre^ mice for primary culture as described in our previous studies (Li et al., 2020a; Huang et al., 2023; Li et al., 2023; Huang et al., 2024). The podocytes of different groups of mice were treated with vehicle or palmitic acid (PA), a prototype free fatty acid increased during obesity, for 24 hours. As shown in **Figures 4A, B**, confocal microscopy demonstrated that PA stimulation significantly increased colocalization of NLRP3 and ASC in WT/WT podocytes, suggesting activation of NLRP3 inflammasome. In podocytes of Smpd1^trg^/Podo^cre^ mice, Smpd1 gene overexpression enhanced PA-induced NLRP3 inflammasome activation. On the contrary, NLRP3 inflammasome formation was prevented by Smpd1 gene deletion in Smpd1^-/-^ podocytes. By dual staining of GSDMD-NT and membrane structure (WGA), we found remarkable elevation of GSDMD-NT on plasma membrane of PA-treated WT/WT podocytes compared with control cells, suggesting GSDMD pore formation on plasma membrane. The PA-induced GSDMD pore formation on plasma membrane was inhibited by Smpd1 gene deletion in Smpd1^-/-^ podocytes but exaggerated by Smpd1 gene overexpression in podocytes of Smpd1^trg^/Podo^cre^ mice (**Figures 4C, D**). Functionally, the release of IL-1β from WT/WT podocytes was significantly increased by PA treatment, which was amplified by Smpd1 gene overexpression but attenuated by Smpd1 gene deletion (**Figure 4E**).

**Figure 5:**
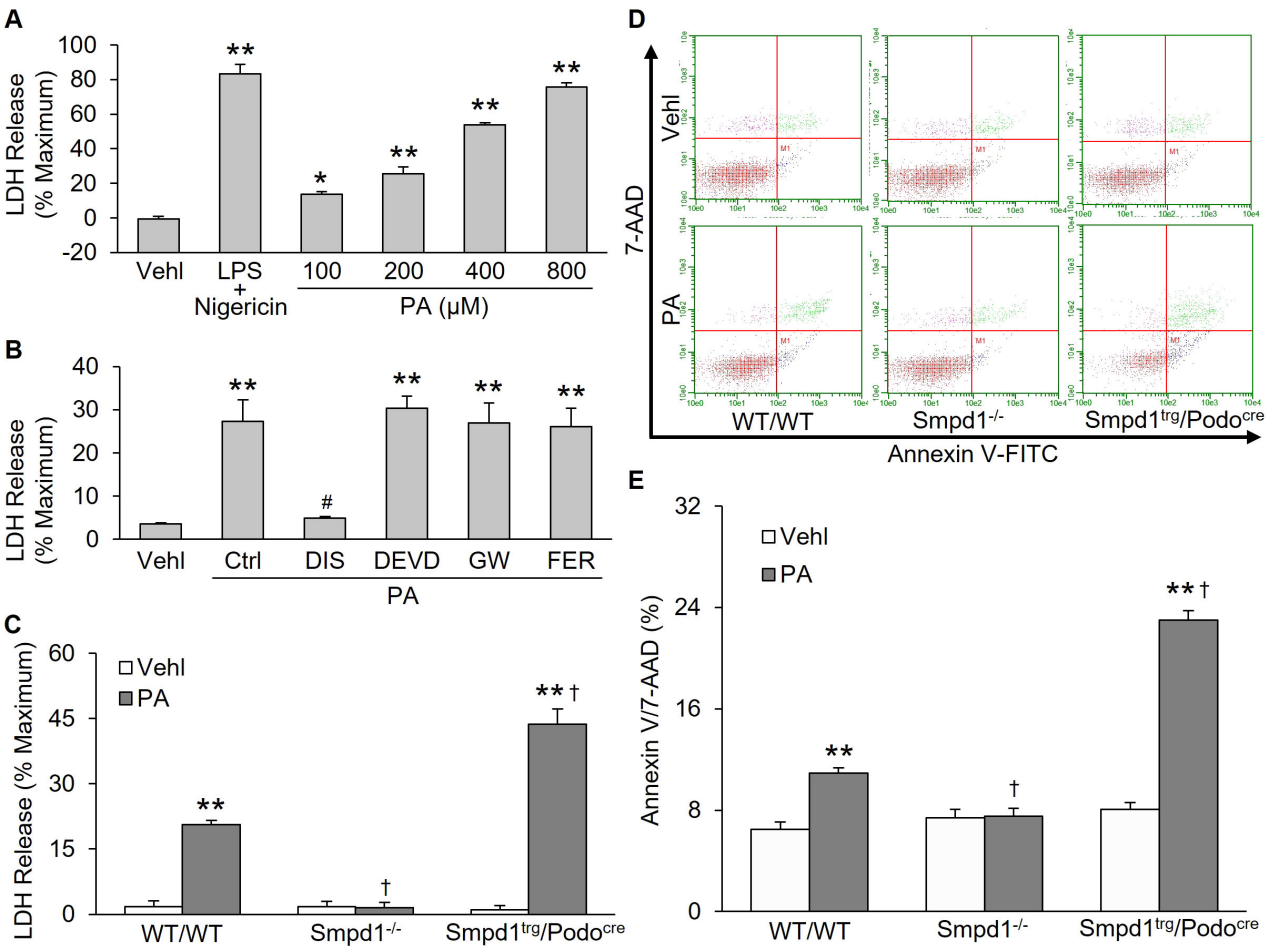
Attenuation of obesity-induced glomerular injury by inhibition of GSDMD pore formation. **(A)** Representative images showing the glomerular expression of podocin in different groups of mice. **(B)** Summarized data showing the glomerular expression of podocin in different groups of mice (n=4). **(C)** Representative images showing the glomerular morphological changes of different groups of mice. **(D)** Summarized data showing the glomerular damage index of different groups of mice (n=5-6). * P<0.05 *vs.* WT/WT group. # P<0.05 *vs.* ND group. † P<0.05 *vs.* Vehl group. ND, normal diet; HFD, high-fat diet; Vehl, vehicle; AMI, amitriptyline; MCC, MCC950; DIS, disulfiram; GDI, glomerular damage index.

To examine PA-induced pyroptosis in podocytes, LDH cytotoxicity assay and flow cytometric analysis of pyroptotic cell death after dual staining of Annecin V-FITC and 7-AAD (7-Aminoactinomycin D) were performed. As shown in **Figure 5A**, PA induced LDH release from podocytes in a dose-dependent manner after 24 hours of treatment at 100, 200, 400, and 800 µM. LPS plus nigericin was included as a positive control for canonical pyroptosis and induced robust LDH release (He et al., 2015). Although higher concentrations of PA caused greater LDH release, 200 µM PA was used for subsequent mechanistic studies because this concentration produced significant pyroptosis while better mimicking obesity-associated lipotoxic stress (Ku et al., 2019; Wajngarten and Silva, 2019; Chobufo et al., 2020; Poznyak et al., 2022). We next determined whether PA-induced pyroptosis was mediated by GSDMD pore formation or by other lytic cell death pathways. Podocytes were pretreated with disulfiram (DIS, 20 µM), an inhibitor of GSDMD pore formation (Hu et al., 2020); Z-DEVD-FMK (DEVD, 20 µM), a caspase-3 inhibitor used to assess GSDME-mediated secondary necrosis (Shen et al., 2021); GW806742X (GW, 1 µM), an MLKL inhibitor used to assess necroptosis (Hao et al., 2022); or Ferrostatin-1 (FER, 1 µM), an inhibitor of ferroptosis-associated membrane damage (Wang et al., 2023). As shown in **Figure 5B**, PA-induced LDH release was markedly attenuated by disulfiram, whereas Z-DEVD-FMK, GW806742X, or Ferrostatin-1 did not significantly affect PA-induced LDH release. These findings suggest that PA-induced pyroptosis in podocytes is primarily mediated by GSDMD pore formation rather than GSDME-mediated secondary necrosis, MLKL-dependent necroptosis, or ferroptosis-associated membrane damage. We further assessed the role of ASM activity in PA-induced podocyte pyroptosis. As shown in **Figure 5C**, PA significantly increased LDH release from WT/WT podocytes, suggesting pyroptotic cell death occurring in podocytes treated with PA. PA-induced pyroptosis was prevented by Smpd1 gene deletion in Smpd1^-/-^ podocytes but remarkably exaggerated by Smpd1 gene overexpression in podocytes of Smpd1^trg^/Podo^cre^ mice. Correspondingly, flow cytometric analysis showed that the ratio of pyroptotic podocytes (dual positive of Annecin V-FITC and 7-AAD stainings) was significantly elevated by PA stimulation in WT/WT podocytes. PA-induced pyroptosis was remarkably exacerbated by Smpd1 gene overexpression in podocytes of Smpd1^trg^/Podo^cre^ mice, while Smpd1^-/-^ podocytes did not show any such increase (**Figures 5D**, **E**). Together with the GSDMD-NT membrane localization shown in **Figure 4**, these data indicate that PA induces GSDMD-mediated pyroptosis in podocytes, which is promoted by ASM activity.

### ASM overexpression impairs lysosome-dependent autophagic flux and promotes GSDMD-NT accumulation in podocytes

**Figure 6:**
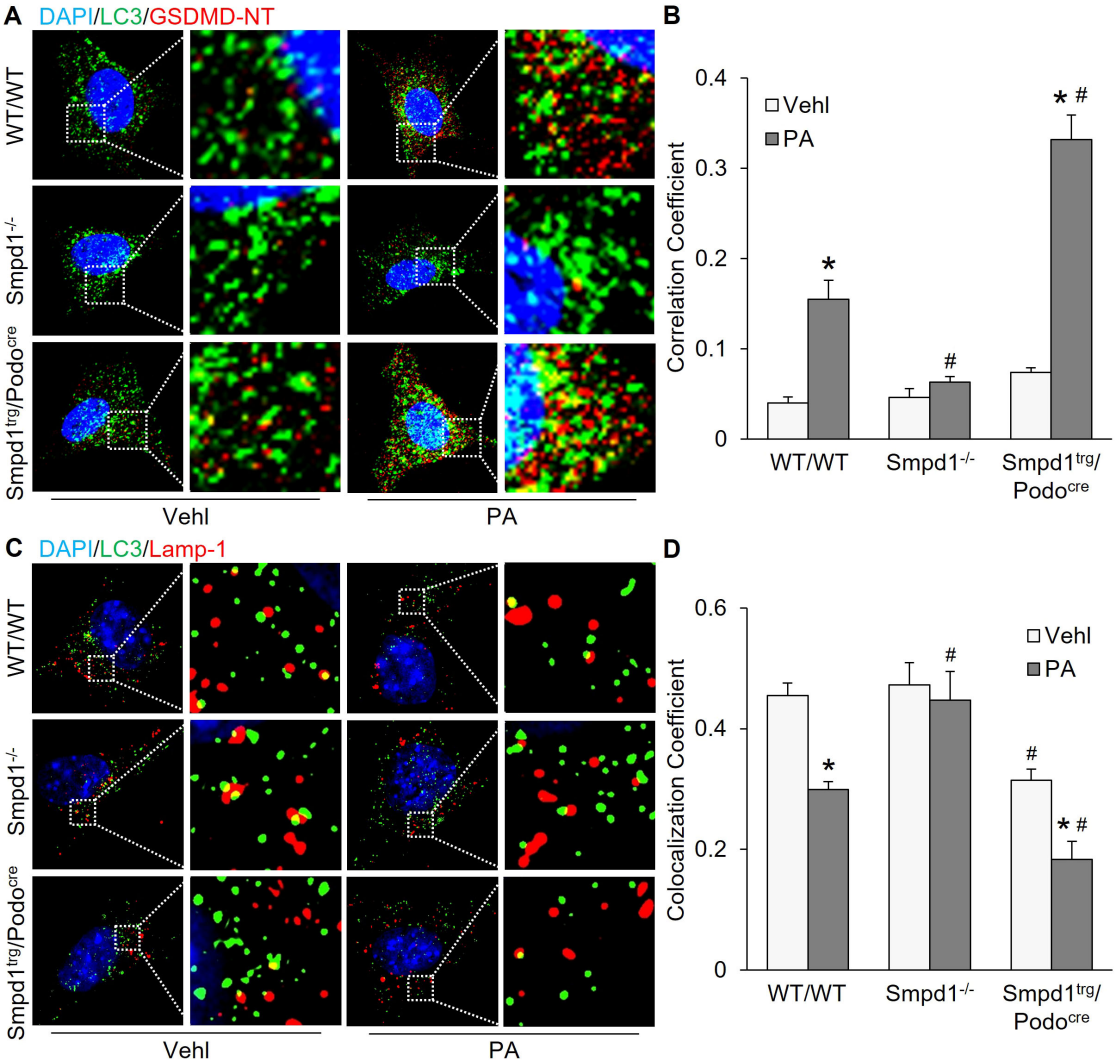
ASM overexpression promotes GSDMD-NT accumulation in autophagosomes and impairs autophagosome–lysosome interaction. **(A)** Representative images showing the colocalization of LC3 and GSDMD-NT in different groups of podocytes. DAPI was used to stain nuclei. **(B)** Summarized data showing the colocalization of LC3 and GSDMD-NT in different groups of podocytes (n=4). **(C)** Representative images showing the colocalization of LC3 and Lamp-1 in different groups of podocytes. DAPI was used to stain nuclei. **(D)** Summarized data showing the colocalization of LC3 and Lamp-1 in different groups of podocytes (n=4). * P<0.05 *vs.* Vehl group. # P<0.05 *vs.* WT/WT group. Vehl, vehicle; PA, palmitic acid; GSDMD-NT, gasdermin D N-terminal.

We next examined whether ASM regulates lysosome-dependent autophagic handling of GSDMD-NT in podocytes, given that the ASM–ceramide signaling pathway has been reported to regulate lysosomal function in podocytes (Huang et al., 2022; Huang et al., 2023). Confocal microscopy showed that PA stimulation significantly increased the colocalization of GSDMD-NT with LC3-positive autophagosomal structures in WT/WT podocytes, suggesting accumulation of GSDMD-NT in autophagosome-associated compartments. In Smpd1^-/-^ podocytes, PA did not significantly alter GSDMD-NT/LC3 colocalization. Conversely, Smpd1 overexpression markedly enhanced PA-induced GSDMD-NT accumulation in LC3-positive compartments (**Figures 6A, B**).

Because increased GSDMD-NT/LC3 colocalization alone does not distinguish enhanced autophagosomal sequestration from impaired autophagic degradation, we next examined autophagosome–lysosome interaction by assessing LC3 and Lamp-1 colocalization using super-resolution microscopy. Under basal conditions, WT/WT podocytes exhibited substantial LC3/Lamp-1 colocalization, consistent with intact autophagosome–lysosome interaction. PA stimulation significantly reduced LC3/Lamp-1 colocalization in WT/WT podocytes. This PA-induced disruption was prevented by Smpd1 gene deletion, whereas Smpd1 overexpression further exacerbated the reduction in LC3/Lamp-1 colocalization (**Figures 6C, D**). These results suggest that ASM overexpression promotes GSDMD-NT accumulation in autophagosomal compartments by impairing lysosome-dependent autophagic flux.

### ASM overexpression suppresses TRPML1-mediated lysosomal Ca^2+^ release and lysosome trafficking in podocytes

**Figure 7:**
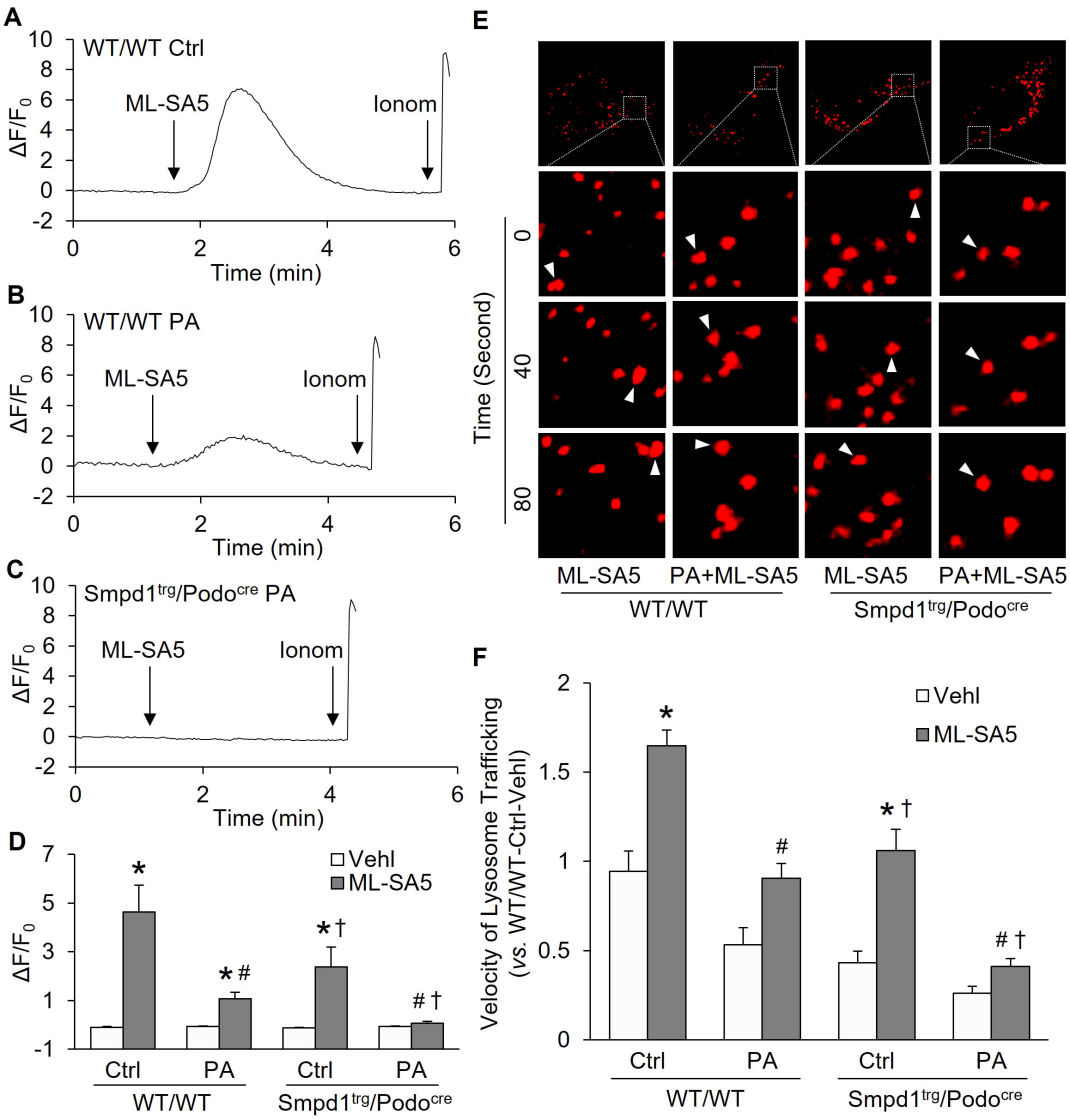
ASM overexpression suppresses TRPML1-dependent lysosomal Ca^2+^ release and lysosome trafficking in podocytes. **(A)** A representative curve showing that ML-SA5 induced elevation of GCaMP3 signal in WT/WT podocytes. **(B)** A representative curve showing that ML-SA5 induced smaller elevation of GCaMP3 signal in WT/WT podocytes treated with PA compared with control cells. **(C)** A representative curve showing that ML-SA5 failed to induce elevation of GCaMP3 signal in podocytes of Smpd1^trg^/Podo^cre^ mice after treatment with PA. **(D)** Summarized data of GCaMP3 fluorescence as the indicator of lysosomal TRPML1 channel activity in different groups of podocytes (n=4-6). **(E)** Representative images showing the lysosome movement in podocytes of WT/WT and Smpd1^trg^/Podo^cre^ mice under different conditions. **(F)** Summarized data showing velocity of lysosome trafficking in podocytes of WT/WT and Smpd1^trg^/Podo^cre^ mice under different conditions (n=3-4). * P<0.05 *vs.* Vehl group. # P<0.05 *vs.* Ctrl group. † P<0.05 *vs.* WT/WT group. Ctrl, control; Vehl, vehicle; PA, palmitic acid; Ionom, ionomycin.

Given the critical role of TRPML1 channel activity in lysosomal trafficking and lysosome–autophagosome interactions in podocytes (Li et al., 2023), we next examined whether TRPML1-mediated Ca^2+^ release contributes to ASM-dependent regulation of lysosomal function and autophagic flux in these cells. To selectively evaluate Ca^2+^ release through lysosomal TRPML1 channels, podocytes were nucleofected with a GCaMP3-ML1 construct, which encodes the genetically encoded Ca^2+^ indicator GCaMP3 fused to the cytoplasmic N-terminus of TRPML1, as previously described (Li et al., 2019; Li et al., 2020a; Li et al., 2021; WHO, 2021). Live-cell fluorescence imaging was performed to continuously monitor changes in GCaMP3 fluorescence intensity (F470), serving as an indicator of TRPML1-mediated Ca^2+^ release. In WT/WT podocytes, the TRPML1 agonist ML-SA5 induced a rapid increase in GCaMP3 fluorescence, followed by a robust response to ionomycin as a positive control. PA treatment significantly reduced ML-SA5-induced GCaMP3 fluorescence in WT/WT podocytes. This PA-induced suppression of TRPML1-mediated Ca^2+^ release was further aggravated in podocytes from Smpd1^trg^/Podo^cre^ mice (**Figures 7A–D**).

To further determine whether these changes were associated with altered lysosomal function, we performed live-cell imaging of lysosome trafficking in podocytes loaded with dextran–Alexa Fluor 555. Lysosome movement was continuously recorded for 10 minutes and analyzed by manual tracking. ML-SA5 increased lysosome movement in WT/WT podocytes, consistent with activation of TRPML1-dependent lysosomal trafficking. PA treatment reduced lysosome movement and blunted the response to ML-SA5. In podocytes from Smpd1^trg^/Podo^cre^ mice, PA-induced impairment of lysosome trafficking was further exacerbated (**Figures 7E, F**). These data indicate that ASM overexpression suppresses TRPML1-mediated lysosomal Ca^2+^ release and lysosome trafficking, providing a mechanistic basis for impaired autophagic flux and GSDMD-NT accumulation in PA-treated podocytes.

### Regulation of lysosome-autophagosome interaction and GSDMD pore formation by TRPML1 channel in podocytes

**Figure 8:**
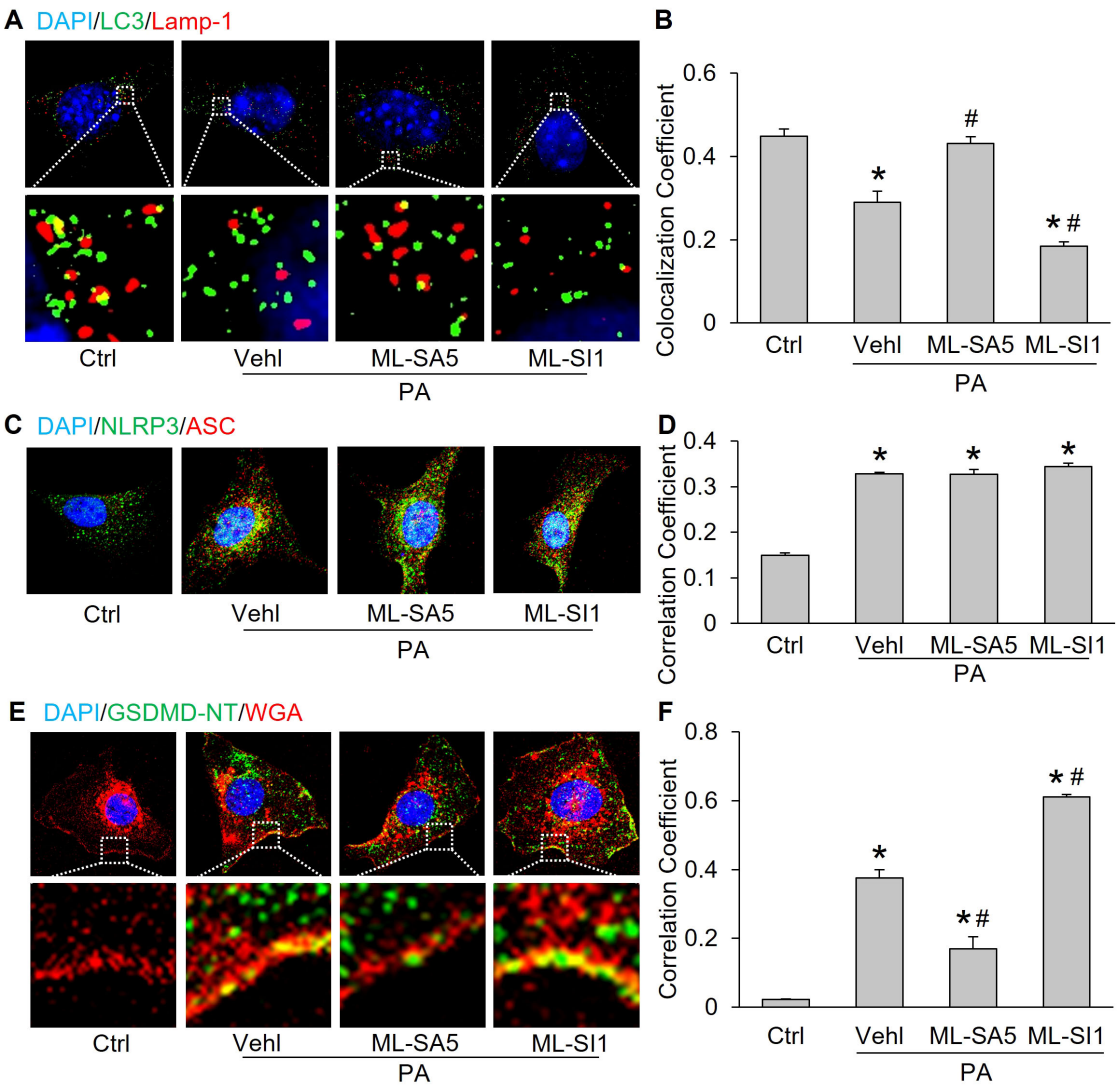
Regulation of lysosome-autophagosome interaction and GSDMD pore formation by TRPML1 channel in podocytes. **(A)** Representative images showing the colocalization of LC3 and Lamp-1 in different groups of podocytes. DAPI was used to stain nuclei. **(B)** Summarized data showing the colocalization of LC3 and Lamp-1 in different groups of podocytes (n=3). **(C)** Representative images showing the colocalization of NLRP3 and ASC in different groups of podocytes. DAPI was used to stain nuclei. **(D)** Summarized data showing the colocalization of NLRP3 and ASC in different groups of podocytes (n=3). **(E)** Representative images showing the colocalization of GSDMD-NT and WGA in different groups of podocytes. DAPI was used to stain nuclei. **(F)** Summarized data showing the colocalization of GSDMD-NT and WGA on plasma membrane of different groups of podocytes (n=3-4). * P<0.05 *vs.* Ctrl group. # P<0.05 *vs.* Vehl group. Ctrl, control; Vehl, vehicle; PA, palmitic acid.

Mechanistically, we tested whether pharmacological manipulation of TRPML1 channel activity can affect lysosome-autophagosome interaction and GSDMD pore formation in podocytes. By super-resolution microscopy, we found that PA-induced reduction in lysosome-autophagosome interaction of WT/WT podocytes was prevented by pre-treatment with a TRPML1 channel agonist, ML-SA5 (1 µM) (Li et al., 2023). On the contrary, pre-treatment with a TRPML1 channel inhibitor, ML-SI1 (20 μM) (Li et al., 2023) significantly exaggerated the downregulation of lysosome-autophagosome interaction by PA in WT/WT podocytes (**Figures 8A, B**). Confocal microscopy of NLRP3 and ASC revealed that PA-induced NLRP3 inflammasome activation in WT/WT podocytes was not altered by pre-treatment with ML-SA5 or ML-SI1 (**Figures 8C, D**). Interestingly, pre-treatment with ML-SA5 remarkably attenuated PA-induced GSDMD pore formation on plasma membrane of WT/WT podocytes. On the contrary, PA-induced GSDMD pore formation on plasma membrane of WT/WT podocytes was significantly reduced by pre-treatment with ML-SI1 (**Figures 8E, F**). These findings suggest that amplification of TRPML1 channel function can attenuate GSDMD pore formation despite the presence of upstream NLRP3 inflammasome activation in podocytes.

## Discussion

Obesity is increasingly recognized as a chronic inflammatory condition characterized by persistent oxidative stress and sterile inflammation, which together promote glomerular injury and accelerate the progression toward chronic kidney disease and end-stage renal disease (Matsuzawa-Nagata et al., 2008; Marseglia et al., 2014; McMurray et al., 2016; Alcala et al., 2017; Bayliak et al., 2019). Substantial evidence has established that oxidative activation of the NLRP3 inflammasome in podocytes represents an initiating event in obesity-related glomerulopathy (ORG), leading to the generation of IL-1β, IL-18, and HMGB1 and triggering local inflammatory amplification within the glomerulus (Cruz et al., 2007; Halle et al., 2008; Martinon et al., 2009; Nour et al., 2009; Abais et al., 2013; He et al., 2015; Boini et al., 2016). However, because inflammasome products are generated in the cytosol rather than through the classical ER–Golgi secretory pathway, the mechanisms governing their release from podocytes and their transition from inflammatory signaling to podocyte death have remained poorly understood. Recent studies in immune and nonimmune cells have identified gasdermin D (GSDMD) pore formation as a critical conduit for inflammasome product release and a decisive executor of pyroptotic cell death following inflammasome activation (He et al., 2015; Dubois et al., 2019; Gao et al., 2021; Tsuchiya et al., 2021), providing a conceptual framework for understanding how inflammasome activation may translate into structural glomerular injury.

In this study, we have demonstrated that GSDMD pore formation is robustly induced in podocytes during obesity and serves as a pivotal mediator linking NLRP3 inflammasome activation to pyroptotic podocyte loss and glomerulosclerosis. While prior reports have documented increased GSDMD cleavage and pyroptosis in podocytes in glomerular diseases such as APOL1-associated podocytopathy, lupus nephritis, membranous nephropathy, and diabetic nephropathy (Cao et al., 2021; Cheng et al., 2021; Wu et al., 2021a; Lv et al., 2022), the present work provides the first evidence that GSDMD pore formation is dynamically regulated in podocytes during obesity and critically determines the severity of ORG. Importantly, our findings reveal that pharmacological inhibition of GSDMD pore formation effectively prevents podocyte pyroptosis and glomerular injury despite persistent upstream NLRP3 inflammasome activation and GSDMD-NT generation, indicating that GSDMD pore formation represents a terminal and targetable checkpoint in obesity-induced podocyte injury. In this regard, exosome secretion from podocytes has been reported to mediate the release of NLRP3 inflammasome products from podocytes to the extracellular space for the initiation of glomerular inflammation and injury under pathological conditions, such as obesity and hyperhomocysteinemia (Huang et al., 2021; WHO, 2021; Huang et al., 2022; Huang et al., 2023; Huang et al., 2024). Our findings in the present study suggest that GSDMD pore and exosome may simultaneously regulate the release of NLRP3 inflammasome products from podocytes to the extracellular space during inflammatory glomerular diseases. Although our data support GSDMD pore formation based on GSDMD-NT plasma membrane localization, IL-1β release, LDH release, Annexin V-FITC/7-AAD-positive pyroptotic cell death, and inhibition by disulfiram, we acknowledge that direct biochemical detection of GSDMD oligomerization, membrane-inserted GSDMD-NT, or high-resolution visualization of GSDMD pores would provide additional mechanistic evidence. Future studies using GSDMD oligomerization assays, membrane fractionation, or super-resolution/electron microscopy will be valuable to further define GSDMD pore assembly in palmitic acid (PA)-treated podocytes.

A major novel insight from this study is the identification of lysosome-dependent autophagic degradation of GSDMD-NT as a key mechanism restraining GSDMD pore formation in podocytes. Autophagy is essential for podocyte homeostasis, and impairment of autophagic flux has been implicated in multiple glomerular diseases (Li et al., 2015; Li et al., 2020c; Li et al., 2023). However, its role in regulating pyroptotic execution has not been previously defined. Our data demonstrate that obesity-related lipotoxic stress promotes GSDMD cleavage and sequestration of GSDMD-NT into autophagosomes while simultaneously inhibiting lysosome–autophagosome fusion, resulting in defective autophagic flux and accumulation of pore-forming GSDMD-NT. This mechanism is consistent with emerging evidence that lysosomes actively regulate GSDMD pore fate by degrading cytosolic GSDMD-NT to prevent excessive pore formation and the transition from a hyperactivated inflammatory state to lytic pyroptosis (Liao et al., 2021). Thus, impaired lysosomal clearance of GSDMD-NT in podocytes provides a mechanistic explanation for sustained pore formation and pyroptotic cell death during obesity. Interestingly, lysosome-dependent degradation of multivesicular bodies has also been reported to control the release of exosomes containing NLRP3 inflammasome products in podocytes under pathological conditions (Li et al., 2021; WHO, 2021; Huang et al., 2022; Huang et al., 2023). The regulation of both GSDMD pore formation and inflammatory exosome release by lysosomes suggests that lysosome function may determine the progression of ORG and other inflammatory glomerular diseases.

Our findings further establish acid sphingomyelinase (ASM) as an important upstream determinant of the lysosome–autophagy–GSDMD axis in podocytes. ASM overactivation and ceramide accumulation have been previously implicated in obesity-induced glomerular injury through ROS generation and NLRP3 inflammasome activation (Boini et al., 2010b; Boini et al., 2014; Boini et al., 2016). In addition, ASM has been shown to promote endothelial NLRP3 inflammasome activation during hypercholesterolemia, and the broader crosstalk between ASM signaling and inflammasome activation has been summarized in previous studies (Koka et al., 2017; Li et al., 2019). ASM/ceramide signaling may activate the NLRP3 inflammasome through several converging stress pathways. Ceramide-enriched lipid raft clustering can facilitate NADPH oxidase assembly and superoxide production, thereby promoting redox-dependent inflammasome activation (Zhang et al., 2007; Boini et al., 2010a; Boini et al., 2011; Boini et al., 2016). Mitochondrial ROS and mitochondrial dysfunction are also well-established triggers of NLRP3 activation (Nakahira et al., 2011), while lysosomal damage and cathepsin release provide another mechanism linking defective lysosomal homeostasis to inflammasome activation (Kim et al., 2008). In addition, altered Ca^2+^ signaling, membrane trafficking, and membrane repair may further contribute to NLRP3 activation under lipotoxic stress. Ceramide has also been shown to promote GSDMD cleavage and pyroptosis in endothelial cells (Liu et al., 2022), suggesting that sphingolipid metabolism may broadly sensitize cells to pyroptotic injury. Extending these observations, our study demonstrates that podocyte-specific ASM overexpression profoundly disrupts lysosomal function, suppresses autophagic flux, and accelerates GSDMD pore formation and pyroptosis, whereas Smpd1 gene deletion preserves lysosome–autophagosome interaction and prevents GSDMD-NT accumulation. Thus, ASM may promote podocyte pyroptosis through two related mechanisms: upstream activation of the NLRP3 inflammasome and downstream impairment of lysosome-dependent autophagic handling of GSDMD-NT. We acknowledge that the present study did not determine the relative contribution of ROS generation, mitochondrial dysfunction, Ca^2+^ signaling, lipid raft formation, membrane trafficking, or membrane repair to ASM-dependent NLRP3 inflammasome activation. Future studies using targeted inhibitors or genetic approaches directed at these individual pathways will be required to define how ASM mechanistically promotes NLRP3 activation upstream of GSDMD cleavage during obesity-related podocyte injury.

Mechanistically, our data identify lysosomal TRPML1 channel-mediated Ca^2+^ release as an important downstream target of ASM-dependent lysosomal dysfunction that governs lysosome trafficking and lysosome–autophagosome interaction in podocytes. TRPML1-dependent Ca^2+^ release is essential for lysosomal mobility and vesicle fusion (Li and Li, 2021). In the present study, PA markedly inhibited TRPML1-mediated Ca^2+^ release in podocytes, and this inhibition was further exacerbated by podocyte-specific Smpd1 overexpression. Consistently, PA impaired lysosome movement and reduced lysosome–autophagosome interaction, whereas activation of TRPML1 restored lysosome trafficking, improved lysosome–autophagosome interaction, and markedly suppressed GSDMD pore formation without altering upstream NLRP3 inflammasome activation. These findings suggest that TRPML1-mediated lysosomal Ca^2+^ signaling mainly controls the autophagic handling and execution phase of pyroptosis rather than upstream inflammasome activation in podocytes during obesity-related lipotoxic stress. Although the present study did not directly examine whether ASM or ceramide alters TRPML1 localization, channel gating, or lysosomal membrane organization in PA-treated podocytes, our previous studies have shown that altered sphingolipid metabolism resulting from acid ceramidase deficiency or ASM overexpression suppresses TRPML1 channel activity, leading to lysosomal trafficking failure and accumulation of autophagosomes and multivesicular bodies in podocytes (Li et al., 2020a; Huang et al., 2021; Huang et al., 2022; Li et al., 2023). These previous studies used GCaMP3-ML1 Ca^2+^ imaging and whole-lysosome patch-clamp recording to establish the functional regulation of lysosomal TRPML1 channels by ASM-associated sphingolipid signaling. In addition, endogenously produced reactive oxygen species (ROS) have been reported to inhibit TRPML1 channel activity and impair lysosome function in podocytes (Li et al., 2021). Given that ASM-dependent ceramide production contributes to lipid raft clustering and NADPH oxidase-dependent superoxide production (Zhang et al., 2007; Boini et al., 2010a; Boini et al., 2011; Boini et al., 2016), ASM overexpression and associated ceramide accumulation may inhibit TRPML1 activity through both sphingolipid-dependent changes in lysosomal membrane signaling and ROS-dependent mechanisms. Further studies using TRPML1 localization analysis, lysosomal membrane lipid profiling, and whole-lysosome electrophysiology in PA-treated podocytes will be valuable to define the precise molecular mechanism by which ASM regulates TRPML1 in obesity-related podocyte injury.

Several limitations should be noted. First, although the present study focuses on podocytes and uses podocyte-specific Smpd1 overexpression mice together with primary podocytes isolated from WT/WT, Smpd1^-/-^, and Smpd1^trg^/Podo^cre^ mice, we cannot exclude the possibility that obesity-induced lysosomal dysfunction also occurs in other renal cell types as part of a broader metabolic stress response. Future studies using cell type-specific markers, single-cell approaches, or cell-specific genetic models will be useful to determine whether the ASM–TRPML1–autophagy–GSDMD pathway is unique to podocytes or shared by other glomerular and tubular cells during obesity. Second, human ORG kidney biopsy samples were not available in the current study. Validation of ASM activation, TRPML1 dysfunction, impaired autophagic flux, GSDMD-NT accumulation, and pyroptotic injury in human ORG samples will be important to establish the translational relevance of this pathway. Third, obesity-associated renal injury may involve multiple overlapping forms of regulated cell death, including pyroptosis, apoptosis, necroptosis, and ferroptosis. In our revised *in vitro* studies, PA-induced LDH release was reduced by the GSDMD pore inhibitor disulfiram, but not by Z-DEVD-FMK, GW806742X, or Ferrostatin-1, suggesting that GSDMD-mediated pyroptosis is the predominant lytic pathway under our experimental conditions. Nevertheless, these pharmacological data do not fully exclude contributions from other cell death pathways *in vivo*. Future studies using pathway-specific genetic models and human ORG samples will be needed to define how pyroptosis interacts with apoptosis, necroptosis, and ferroptosis during obesity-related glomerular injury.

In summary, the present study demonstrates that obesity-induced activation of the ASM–ceramide signaling pathway suppresses TRPML1-mediated lysosomal Ca^2+^ release, disrupts lysosome–autophagosome fusion, and impairs the autophagic degradation of GSDMD-NT. These alterations lead to sustained GSDMD pore formation, pyroptotic loss of podocytes, and progressive glomerulosclerosis. This mechanism provides an integrated framework linking sphingolipid metabolism, lysosomal dysfunction, defective autophagy, and inflammatory cell death in podocytes, offering insight into how chronic low-grade inflammation in obesity progresses to irreversible glomerular injury and FSGS. From a therapeutic standpoint, these findings highlight the potential of targeting autophagic degradation of GSDMD-NT in podocytes as a novel strategy to prevent or treat glomerular inflammation and injury across various pathological conditions, including obesity, hyperhomocysteinemia, and diabetes mellitus.

## Data Availability

The original contributions presented in the study are included in the article/supplementary material. Further inquiries can be directed to the corresponding author.
